# Glabridin, a bioactive component of licorice, ameliorates diabetic nephropathy by regulating ferroptosis and the VEGF/Akt/ERK pathways

**DOI:** 10.1186/s10020-022-00481-w

**Published:** 2022-05-20

**Authors:** Hongtao Tan, Junxian Chen, Yicong Li, Yingshan Li, Yunchang Zhong, Guangzhao Li, Lingling Liu, Yiqun Li

**Affiliations:** 1 Department of Traditional Chinese Medicine, Huizhou Municipal Central Hospital, Huicheng District, No. 41 Eling North Road, Huizhou, 516000 Guangdong China; 2 The First College of Clinical Medicine, Guangzhou University of Chinese Medicine, Guangzhou, China

**Keywords:** Glabridin, Diabetic nephropathy, Bioinformatics, Ferroptosis, VEGF signaling pathway

## Abstract

**Background:**

Glabridin (Glab) is a bioactive component of licorice that can ameliorate diabetes, but its role in diabetic nephropathy (DN) has seldom been reported. Herein, we explored the effect and underlying mechanism of Glab on DN.

**Methods:**

The bioactive component-target network of licorice against DN was by a network pharmacology approach. The protective effect of Glab on the kidney was investigated by a high-fat diet with streptozotocin induced-diabetic rat model. High glucose-induced NRK-52E cells were used for in vitro studies. The effects of Glab on ferroptosis and VEGF/Akt/ERK pathways in DN were investigated in vivo and in vitro using qRT-PCR, WB, and IHC experiments.

**Results:**

Bioinformatics analysis constructed a network comprising of 10 bioactive components of licorice and 40 targets for DN. 13 matching targets of Glab were mainly involved in the VEGF signaling pathway. Glab treatment ameliorated general states and reduced FBG, HOMA-β, and HOMA-insulin index of diabetic rats. The renal pathological changes and the impaired renal function (the increased levels of Scr, BUN, UREA, KIM-1, NGAL, and TIMP-1) were also improved by Glab. Moreover, Glab repressed ferroptosis by increasing SOD and GSH activity, and *GPX4*, *SLC7A11*, and *SLC3A2* expression, and decreasing MDA and iron concentrations, and *TFR1* expression, in vivo and in vitro. Mechanically, Glab significantly suppressed VEGF, p-AKT, p-ERK1/2 expression in both diabetic rats and HG-induced NRK-52E cells.

**Conclusions:**

This study revealed protective effects of Glab on the kidney of diabetic rats, which might exert by suppressing ferroptosis and the VEGF/Akt/ERK pathway.

## Background

In China, there is a projected increase in the prevalence of diabetes mellitus (DM) from 98.4 million in 2013 to 142.7 million in 2035 (Forouhi and Wareham 2014), due to the increasing prevalence of unhealthy lifestyles and obesity, which will increase medical and economic burdens in our country. As one of the microvascular complications of DM, diabetic nephropathy (DN) will cause irreversible damage to kidney tissues, which is responsible for about 50% of cases of end-stage renal disease (ESRD) (Alicic et al. 2017; Shlipak 2010). Patients with DN often manifest different degrees of persistent proteinuria. It has been reported that about 30% of DM patients will develop proteinuria without intervention (Lang and Shlipak 2016). The angiotensin-converting-enzyme inhibitor is the first treatment recommended for proteinuric patients as it could reduce proteinuria levels and slow the progression of DN (Gross et al. 2005; Lim 2014). However, their clinical applications are limited due to their effects of lowering blood pressure and decreasing oxygen demand in the heart (Sidorenkov and Navis 2014). Combining with that the progression to ESRD is irreversible, discovering novel renoprotective drugs for delaying renal damage progress is still extremely important to DN therapy.

As a precious deposit for human health care, traditional Chinese medicine (TCM) has long been utilized in improving life quality and curing ailments, including DM and its complications, in China (Zhang and Jiang 2012). The discovery of artemisinin for malarial therapy proves that TCM could be an effective strategy even against the most challenging health issues (Tu 2011). Up till now, about 90 Chinese patent medicines for DM therapy have been certificated by National Medical Products Administration. Notably, approximately one-fifth of these medicines contain licorice (Jiang et al. 2020). Based on the theory of TCM, licorice confers functions of boosting Qi and nourishing Yin, clearing heat, and promoting fluid production, which is commonly utilized in improving breath, enhancing strength, and alleviating dry skin, as well as relieving hungry for DM patients. Increasing studies have shown that licorice extract ameliorates DN and improves renal function by regulating the cellular processes involved in DN (Kataya et al. 2011; Thakur et al. 2017). However, the material bases and mechanisms involved in the role of licorice against DN are largely elusive.

As known, oxidative stress is another causative factor in addition to hyperglycemia for driving DN progression (Liang et al. 2017). Cystine/glutamate transporter system (${\text{X}}_{{\text{C}}}^{. - }$)/glutathione peroxidase-4 (GPX4) is a critical anti-oxidant system for the reduction of oxidative stress, which is also the key mediator of ferroptosis (Dixon and Stockwell 2014). Ferroptosis is an iron-dependent cell death with a characteristic of lipid peroxidation. In recent years, mounting studies signposted a vital role of ferroptosis in DN progression, supporting that ferroptosis could be a potential therapeutic target for DN (Wang et al. 2020; Li et al. 2021). The anti-oxidant effects of licorice extract have been widely reported in researches on various types of diseases (Chin et al. 2007). Moreover, recent studies revealed the function of licorice extract in inhibiting ferroptosis in several diseases (Wang et al. 2019; Wen et al. 2021); while ferroptosis is a target of licorice extract in DN is not documented so far.

In this study, network pharmacology analysis was conducted to preliminarily uncover the bioactive component and targets on the action of licorice related to DN. Based on the above analysis, Glabridin (Glab) was chosen for further analysis to investigate its effects and the mechanisms on DN in vivo and in vitro. This research is expected to lay a scientific foundation of the action of licorice for ameliorating DN, and provide a new way for the treatment of DN.

## Methods

### Bioinformatics analysis

#### Bioactive component screening and target prediction

Traditional Chinese Medicine System Pharmacology Database (TCMSP) is a unique system pharmacology platform of TCM, which can provide the relationship between drugs, targets and diseases. All the chemical compounds’ data of licorice were collected from TCMSP (http://lsp.nwu.edu.cn/tcmsp.php) (Ru et al. 2014), and the bioactive components were obtained for further analysis based on a screening threshold of oral bioavailability (OB) ≥ 50% and drug-likeness (DL) ≥ 0.4. The two-dimensional (2D) structures of all of the identified components were downloaded from PubChem Compound Database (https://www.ncbi.nlm.nih.gov/pccompound).

Three databases, which included GeneCards (http://www.genecards.org/) (Stelzer et al. 2016), BATMAN-TCM (http://bionet.ncpsb.org/batman-tcm) (Liu et al. 2016), and STITCH database (http://stitch.embl.de/) (Kuhn et al. 2008), were combined to predict relevant targets of bioactive components from licorice comprehensively. GeneCards database automatically integrates gene-centric data from approximately 125 web sources, while BATMAN-TCM ranks potential drug-target interactions based on their similarity to the known drug-target interactions. STITCH database integrates many sources of experimental and manually curated evidence with text-mining information and interaction predictions. The targets were finally obtained from the aforementioned three tools after eliminating duplications and unified names.

#### Potential targets of DN mining

DisGeNET (https://www.disgenet.org/) (Piñero et al. 2017), a useful platform, provides the search of the molecular underpinnings of diseases, the analysis of disease genes, the validation of predicted genes, and so on. In this study, DN-associated target genes were searched with the keywords “Diabetic nephropathy” based on two databases: GeneCards and DisGeNET.

#### Common targets of bioactive component and DN identification

The DN-associated targets obtained from the above two databases and targets of active compounds were submitted to identify licorice active compounds-DN common targets using the “Venn” R package.

#### Bioactive component-target network of licorice against DN construction

Network construction was visualized through Cytoscape version 3.7.0 software (Su et al. 2014) to reveal the relationship between active compounds and potential targets. Nodes indicate the bioactive components and targets, while edges represent the intermolecular interactions between active compounds and targets.

#### Functional enrichment and pathways analysis

To explore characteristic biological relevance and systematic functions attribute of the potential target for licorice in DN, Gene Ontology (GO) functional annotation that comprising of biological process (BP), cell component (CC), and molecular function (MF), and Kyoto Encyclopedia of Genes and Genomes (KEGG) pathway analysis were performed using “clusterProfiler” R package (Yu et al. 2012). GO and KEGG items were ranked and visualized according to the gene count with a cut-off value of p < 0.05.

### Experimental validation

#### Animal experimental procedure

The animal experiment in this study was approved by the Animal Ethics Committee of Huizhou Municipal Central Hospital (Code no. kyll202187). Twenty Sprague–Dawley (SD) rats (220–250 g, male) obtained from provided by Guangdong Medical Laboratory Center (Guangzhou, China) were housed in a room at the constant temperature (22 ± 2) °C with a relative humidity of 50 ± 5% under an alternate 12-h light/dark cycle. All the rats were given ad libitum access to food and water and subjected to acclimatization for a week.

The diabetic rat model was established refer to Furman (Furman 2021) described. Briefly, 15 rats were randomly selected to receive a high-fat diet (HFD), and the remaining rats received a normal diet (ND). After exposing to the respective diets three weeks, the HFD-fed rats were administrated with 40 mg/kg/day streptozotocin (STZ; Sigma-Aldrich, MO, USA; dissolved in 0.1 M citrate buffer) by intraperitoneal injection after overnight fasting for ten consecutive days and DN-fed rats were received the equal volume of the vehicle (0.1 M citrate buffer) only. Blood glucose levels were measured after streptozotocin injection with a glucometer. Only rats with two consecutive morning blood glucose levels ≥ 15 mM were considered diabetic and included in the study. Diabetic rats were then randomly assigned to three groups as follows: DM, DM + Glab, and DM + Rosi (rosiglitazone) groups. For the DM + Glab group, the diabetic rats were given 50 mg/kg Glab via intraperitoneal injection. The diabetic rats in the DM + Rosi group were treated with 5 mg/kg Rosi via intragastric administration. Meanwhile, the control and TM groups were given an equivalent volume of normal saline. Each treatment was administrated for 28 consecutive days.

During the experiment, the body weights, food and water intake of all rats were recorded at 1-week intervals from week 0 to week 4. The urine samples were collected using metabolic cages within 24 h on the day before the end of the experiment. On the last day (day 28), the blood samples were collected from the tail vein after fasted overnight. After anesthetizing and sacrificing rats with pentobarbital, the pancreas and kidney of rats were excised and weighed then stored at − 80 °C for further analysis.

#### Measurement of biochemical parameters and kidney index

After centrifuging the collected blood samples at 3000 rpm for 20 min at 4 °C, the serum was obtained to determine the level of fasting blood glucose (FBG) and fasting insulin (FINS) by glucometer and enzyme-linked immunosorbent assay (ELISA) kit (#RAB0904; Sigma-Aldrich, MA, USA), respectively. The homeostasis model assessments of β-cell function (HOMA-β) and insulin resistance (HOMA-IR) were calculated as follow formulas (Matthews et al. 1985): HOMA-β = 20 × FINS/(FBG-3.5); HOMA-IR = (FINS × FBG)/22.5.

In order to monitor renal function, serum creatinine (Scr), blood urea nitrogen (BUN), urinary albumin excretion rate (UAER), as well as kidney index (kidney index = kidney weight (mg)/body weight (g)) were also measured.

#### Histopathologic examination

Pancreas and kidney tissue samples were immersed in 4% buffered paraformaldehyde overnight for fixation. After dehydration, the fixed tissues were embedded in paraffin and cut into pieces (4 μm sections). To investigate the histopathological change of the pancreas and kidney, the sections were subjected to hematoxylin–eosin (H&E) staining. According to the standard protocols, periodic acid-Schiff (PAS) staining was carried out to observe renal glycogen deposits, and Sirius Red staining was performed for visualization of collagen fibers. The BX51 microscope (Olympus, Tokyo, Japan) was applied to observe and image the histopathological condition of diverse groups.

#### Alloknesis assessment

For testing alloknesis in the DM model rats, scratching behavior was recorded and quantified as reported previously (Cheng et al. 2019). A von Frey filament (0.7 mN) was utilized to elicit scratching responses on the nape of the neck, and the alloknesis score was determined by calculating the total number of scratching reactions.

#### Immunohistochemistry

The paraffin-embedded kidney sections were deparaffinized and hydrated. After an antigen retrieval step, the sections were incubated primary antibodies against VEGF (#ab32152; Abcam, MA, USA), p-AKT (Ser473; #ab81283; Abcam), and p-ERK1/2 (#ab17942; Abcam) overnight at 4 °C, respectively. Subsequently, the sections were incubated with secondary antibody (#ab7090; Abcam) for 2 h, followed by counterstaining with hematoxylin. The positively stained images were observed and analyzed under the BX51 microscope.

#### Western blot

The proteins of urine, kidney, and cells were extracted and analyzed by western blot. After quantifying the concentration using a BCA protein assay kit (Beyotime, Shanghai, China), the equal amount of proteins was separated by SDS-PAGE gel and transferred onto PVDF membranes. Then, the membranes were blocked by skimmed milk for 1 h and incubated with primary antibodies overnight, followed by incubated with secondary antibody for 2 h. Finally, by using an ECL kit (#ab65623; Abcam), the protein bands were visualized, of which intensities were measured by Image J 6.0 software. In addition to antibody against SLC3A2 (#12-0981-81; Thermo Fisher Scientific, MA, USA) the primary antibodies used in this study were all supplied by Abcam, which included anti-KIM-1 (#ab233720), anti-NGAL (#ab216462), anti-TIMP-1 (#ab58425), anti-GPX4 (#ab125066), anti-SLC7A11 (#ab175186), anti-TFR1 (#ab269513), anti-VEGF, anti-Akt (#ab8805), anti-p-AKT (Ser473), anti-ERK1/2 (#ab184699), anti-p-ERK1/2, and anti-β-actin (#ab8226), β-actin was utilized as an internal control.

#### Advanced glycation end products (AGEs) detection

Kidney samples were homogenized and subsequently centrifuged for supernatant collection. The level of AGEs was measured by using an ELISA kit (#STA-817; Cell Biolabs, CA, USA).

#### Assessment of oxidative stress

To observe the effect of Glab on oxidative stress in NP, activities of superoxide dismutase (SOD) and catalase (CAT), contents of glutathione (GSH), malondialdehyde (MDA), and iron, as well as intracellular reactive oxygen species (ROS) were respectively measured by colorimetric SOD activity assay kit (#ab65354; Abcam), CAT assay kit (#CAT100; Sigma-Aldrich), fluorometric GSH assay kit (#ab65322; Abcam), lipid peroxidation (MDA) assay kit (#ab118970; Abcam), iron Assay kit (#ab83366; Abcam), and in vitro ROS/RNS assay kit (#STA-347; Cell Biolabs) following protocols provided by the manufacturers.

#### Quantitative real-time PCR (qRT-PCR)

By using Trizol (Thermo Fisher Scientific), total RNA from the kidneys and NRK-52E cells of diverse groups was extracted to conduct reverse transcription. Afterward, qRT-PCR analysis was performed with SYBR green reagent on the 7500 Real-Time PCR System. The analyzed genes and the primer sequences are listed in Table 1. The efficiency (e) of the PCR reactions was calculated for every primer as the following formula: e = 10^∧^ (1/ − s) − 1, where s is the slope of the standard curve, and only primers with efficiencies between 85 and 115% were used for this study. The mRNA level was normalized to endogenous housekeeping gene β-actin and relative to the calibrator sample using the 2^−ΔΔCt^ method (Livak and Schmittgen 2001). The kidney and NRK-52E cell samples from the control group were used as the calibrator sample for in vivo and in vitro analysis, respectively.

**Table 1:** Primer sequences

Gene	Sequence	
GPX4	Forward (5′–3′)	GGGGACAAAGAGCCGGTAG
	Reverse (5′–3′)	GGTTACTGGGACCTAGGGGA
SLC7A11	Forward (5′–3′)	CAACGCTGTCTCTCACTGGT
	Reverse (5′–3′)	GACTGCCTTGACTTCCGTGA
SLC3A2	Forward (5′–3′)	GGGTCGCCTAGTTTGGAGAG
	Reverse (5′–3′)	GGTCAGGACACACTCACGTT
TFR1	Forward (5′–3′)	GAGCACGTTGGTTCCCTACA
	Reverse (5′–3′)	GGGGTCACAGCTGAAAAGGA
β-actin	Forward (5′–3′)	TTTCCAGCCTTCCTTGGGTATG
	Reverse (5′–3′)	CACTGTGTTGGCATAGAGGTCTTTAC

#### Cell culture

Rat renal tubular epithelial (NRK-52E) cells purchased from the American Type Culture Collection (VA, USA) were cultured in DMEM containing 10% FBS and 1% penicillin–streptomycin mixture at 37 °C in a 5% CO_2_ incubator. High glucose (HG; 30 mM glucose) treatment was performed to induce the condition of DN in the cells; while cells treated with 5.5 mM glucose were considered the control.

#### Cell apoptosis assay

The cell apoptosis was assessed using the Annexin V-PI staining approach. Briefly, after treatment in diverse groups, cells were harvested and washed three times with PBS, followed by resuspended in buffer. Afterward, 200 μL cell suspension was added with 5 μL Annexin V-FITC followed by 10 μL propidium iodide for 10 min incubation in the dark. Finally, apoptosis was analyzed by flow cytometry (Beckman Coulter, CA, USA).

#### Cell viability analysis

The cell viability was detected by Cell Counting Kit-8 (CCK-8; Dojindo, Kumamoto, Japan) following the instructions of the manufacturer. In brief, after treatment in diverse groups, 10% of CCK-8 reagent (v/v) was added to each well of the cells for 1.5 h incubation. Cell viability was detected at 450 nm using a microplate reader (Bio-Rad, CA, USA).

#### Cellular lipid peroxidation accumulation assessment

The lipid peroxidation accumulation of NRK-52E cells was assessed using the C11-BODIPY probe. After treatment, 5 μM BODIPY™ 581/591 C11 (Thermo Fisher Scientific) was added for half an hour incubation at 37◦C. After being pelleted, rinsed, and resuspended in PBS, cells were stained with DAPI and observed under the BX51 microscope.

### Statistical analysis

Data are from three independent experiments measured in triplicate at least, and represented as mean ± SD. Data were analyzed by one-way or two-way analysis of variance followed by Tukey’s post hoc test using Prism 8 software (GraphPad, USA). Differences with p < 0.05 were considered to be statistically significant.

## Results

### Bioinformatics analysis of licorice in diabetic nephropathy

**Fig. 1:**
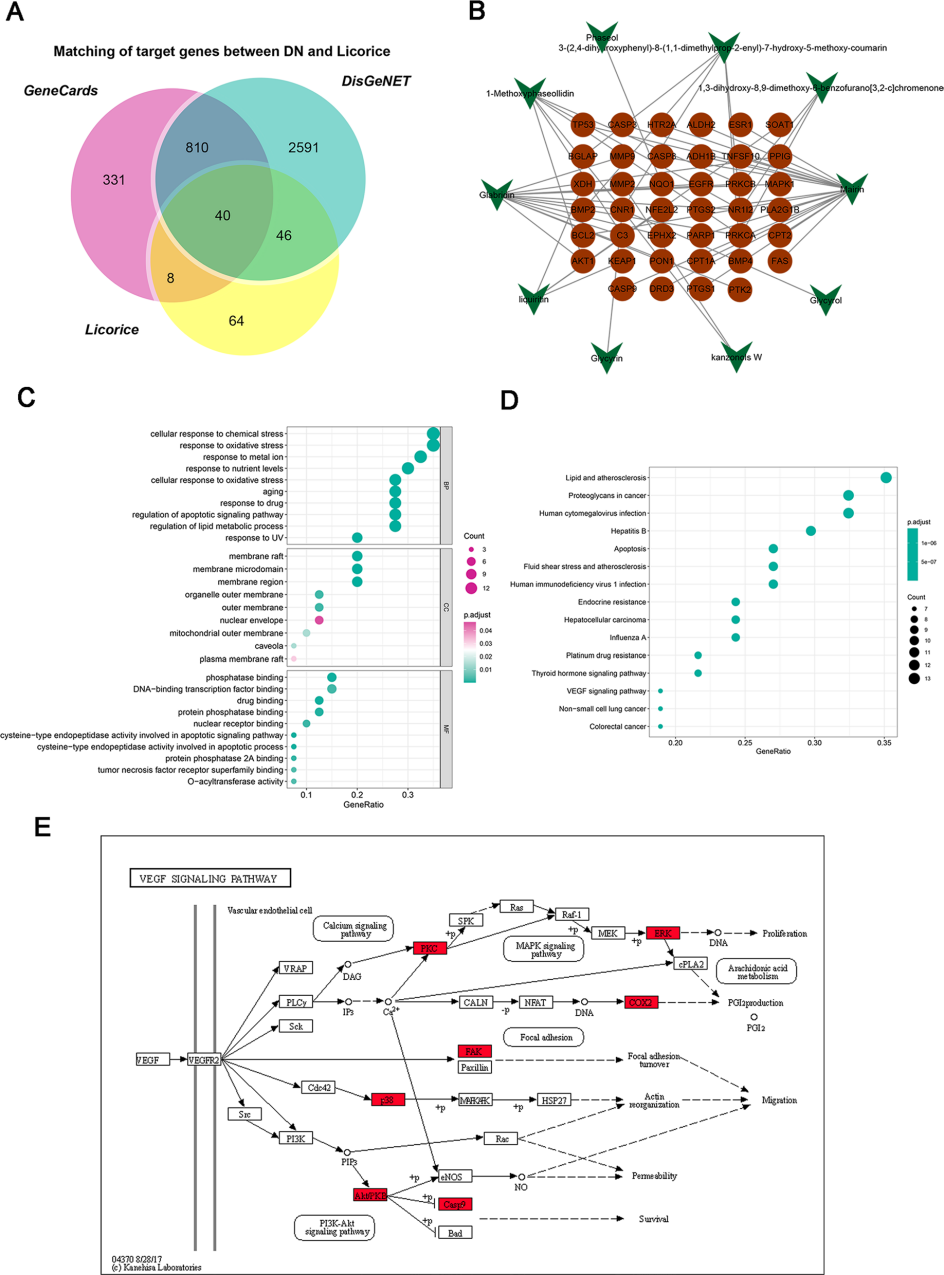
Bioinformatics analysis of licorice in diabetic nephropathy. **A** Common targets between putative predicted targets of licorice and targets associated with DN in the GeneCards and DisGeNET databases. **B** Bioactive component-target network of licorice against DN. The green nodes denote the bioactive components in licorice, and the red nodes denote the corresponding targets of the components. **C** GO and (**D**) KEGG enrichment analysis of 40 targets from the network. **E** Among the 40 targets, the genes in red rectangles were targets of Glab, which were mainly enriched in the VEGF signaling pathway in DN

Based on a threshold of OB ≥ 50% and DL ≥ 0.4, 14 bioactive components of licorice were screened out via the TCMSP databases. Given that the components mainly play pharmacological effects through regulating their target, three predictive tools were applied to predict relevant targets of the 14 bioactive components from licorice. A total of 243 target genes were finally obtained after intersecting the results from three tools. The information of identified bioactive components and their predicted targets were listed in Table 2. Through the GeneCards and DisGeNET databases, the retrieved results were integrated to obtain the DN-associated target genes. Meanwhile, the 243 target genes of licorice were mapped to the DN-associated target genes, and a Venn diagram was drawn. The Venn diagram revealed 40 common targets of the identified bioactive components and DN, which were collected as the potential targets of the protective effect of the identified bioactive components on DN (Fig. 1A).

**Table 2:** The active compound of licorice

Molecule ID	Molecule name	Structure	OB (%)	DL	Targets
MOL000211	Mairin		55.38	0.78	CASP8, TP53, BCL2, CASP3, ANPEP, FAS, MAPK10, PARP1, CASP2, CASP9, CYCS, TOP1, BCL2L1, DIABLO, CCND3, FASLG, MCL1, BIRC5, ANXA5, TNFSF10, ITIH4, GPBAR1, ADRA1A, ADRB2, ADRA2A, ADRA1D, ADRA1B, ADRA2C, DBH, ALDH2, CALM2, CALM3, ADRA2B, CALM1, ABAT, ESRRG, COX6C, COX5B, SLC8A1, COX7C, COX1, F12, AKR1C2, TRPV1, AKR1D1, COX5A, COX3, AR, COX7A1, FECH, COX4I1, PLA2G1B, COX6A2, FADS2, TYR, FADS1, ADH1C, PTGS1, COX6B1, FABP6, CES1, PTGS2, ELOVL4, SRD5A2, COX7B, COX2, GABBR1, COX8A, NR1H4, HTR2A, ADORA2A, ESR1, NR3C1, DRD2, CPT2, HRH1,, KCNH2, HBA2, ATP1A1, KCNN4, DRD3, CPT1A, HBA1, CACNG1, ADH1B, AKR1C1, HMGCR
MOL002311	Glycyrol		90.78	0.67	BCHE, ENDOG, MAOB, CNR2, CNR1, DRD2
MOL004820	Kanzonols W		50.48	0.52	UGT1A1, SULT1A3, SULT2B1, MMP2, CNR2, CNR1, DRD2
MOL004849	3-(2,4-dihydroxyphenyl)-8-(1,1-dimethylprop-2-enyl)-7-hydroxy-5-methoxy-coumarin		59.62	0.43	UGT1A1, NR1I2, UGT1A, MAOA, MAOB, BMP4, BMP2, ALPP, BGLAP, CTRL, NR2, CNR1, DRD2
MOL004856	Gancaonin A		51.08	0.4	CES2
MOL004863	3-(3,4-dihydroxyphenyl)-5,7-dihydroxy-8-(3-methylbut-2-enyl)chromone		66.37	0.41	UGT1A1
MOL004879	Glycyrin		52.61	0.47	CNR2, CNR1, DRD2
MOL004903	Liquiritin		65.69	0.74	NEUROG3, NMUR2, UGT1A1, SULT2B1, KCNJ5, HTR3A, BCHE, MAOB, KEAP1, NFE2L2, XDH, SOAT1, MTTP, SOAT2
MOL004908	Glabridin		53.25	0.47	UGT1A1, MAPK1, CYP2B6, MMP9, MIR148A, CHKA, CYP3A4, EGFR, PON1, SLC6A4, SRC, EPHX2, PTK2, TYR, PPIG, CNR2, CNR1, DRD2, SEC14L3, PPP2CA, PRKCA, NR1I2, ALOX5, PPP2CB, SEC14L2, DGKA, PRKCB, SEC14L4, AKT1, CCR7, ABHD6, MGLL, FCER1G, SUMO1, GPR55, CHRNB2, FCER1A, CAV3, RNF207, C3, DAGLA, PLIN5, ZP3
MOL004914	1,3-dihydroxy-8,9-dimethoxy-6-benzofurano[3,2-c]chromenone		62.9	0.53	BCHE, ACHE, ADPRH, SEC14L3, PPP2CA, PRKCA, NR1I2, ALOX5, PPP2CB, SEC14L2, DGKA, PRKCB, SEC14L4
MOL004959	1-Methoxyphaseollidin		69.98	0.64	CNR2, CNR1, DRD2, SEC14L3, PPP2CA, PRKCA, NR1I2, ALOX5, PPP2CB, SEC14L2, DGKA, PRKCB, SEC14L4, AKT1, CCR7, ABHD6, MGLL, FCER1G, SUMO1, GPR55, CHRNB2, FCER1A, CAV3, RNF207, C3, DAGLA, PLIN5, ZP3
MOL005001	Gancaonin H		50.1	0.78	CES2
MOL005003	Licoagrocarpin		58.81	0.58	UGT1A1
MOL005017	Phaseol		78.77	0.58	CRYZ, VKORC1, NQO1

To understand the interaction relationship between the bioactive components and their corresponding targets, network pharmacology analysis was performed. Using the Cytoscape, a bioactive component-target network of licorice against DN consisting of a total of 50 nodes and 55 edges was constructed, in which the colorized red circles correspond to the targets and the bioactive components calculated in green from licorice. (Fig. 1B). It is interesting that only 10 of 14 bioactive components were contained in this network since another four bioactive components were removed for having no relevant targets. The result showed that MOL000211 (Mairin) and MOL004908 (Glabridin, Glab) have obviously more targets to play a key role in the interaction network, which correspond to 18 and 13 targets, respectively. Thus, they could be defined as the key component behind the effect of licorice against DN. Considering that Glab has been reported to effectively ameliorate DM in previous research, Glab was selected to investigate its role and potential mechanism in protecting the kidney from DN in the subsequent experiments.

To understand the regulatory mechanism behind the potential targets of the identified bioactive components on DN, the functional annotation for the potential targets was performed. GO enrichment revealed that “cellular response to chemical stress”, “response to oxidative stress”, and “response to metal ion” potential targets were the most related biological processes of these potential targets (Fig. 1C). For cellular components, the potential targets of licorice on DN were mainly enriched in “membrane raft”, “membrane microdomain”, and “membrane region” (Fig. 1C). In addition, the targets of molecular function mainly involved “phosphatase binding” “DNA-binding transcription factor binding”, and “drug binding” (Fig. 1C). KEGG pathway analysis showed these potential targets were significantly associated with the pathway of “lipid and atherosclerosis”, “proteoglycans in cancer”, and “human cytomegalovirus infections” (Fig. 1D). Among the potential targets of Glab against DN, 7/13 major nodes were mainly concentrated in the VEGF signaling pathway (Fig. 1E), which suggested the effect of Glab on DN might be exerted by regulating this pathway.

#### The anti-hyperglycemic effect of Glab on DM rats

**Fig. 2:**
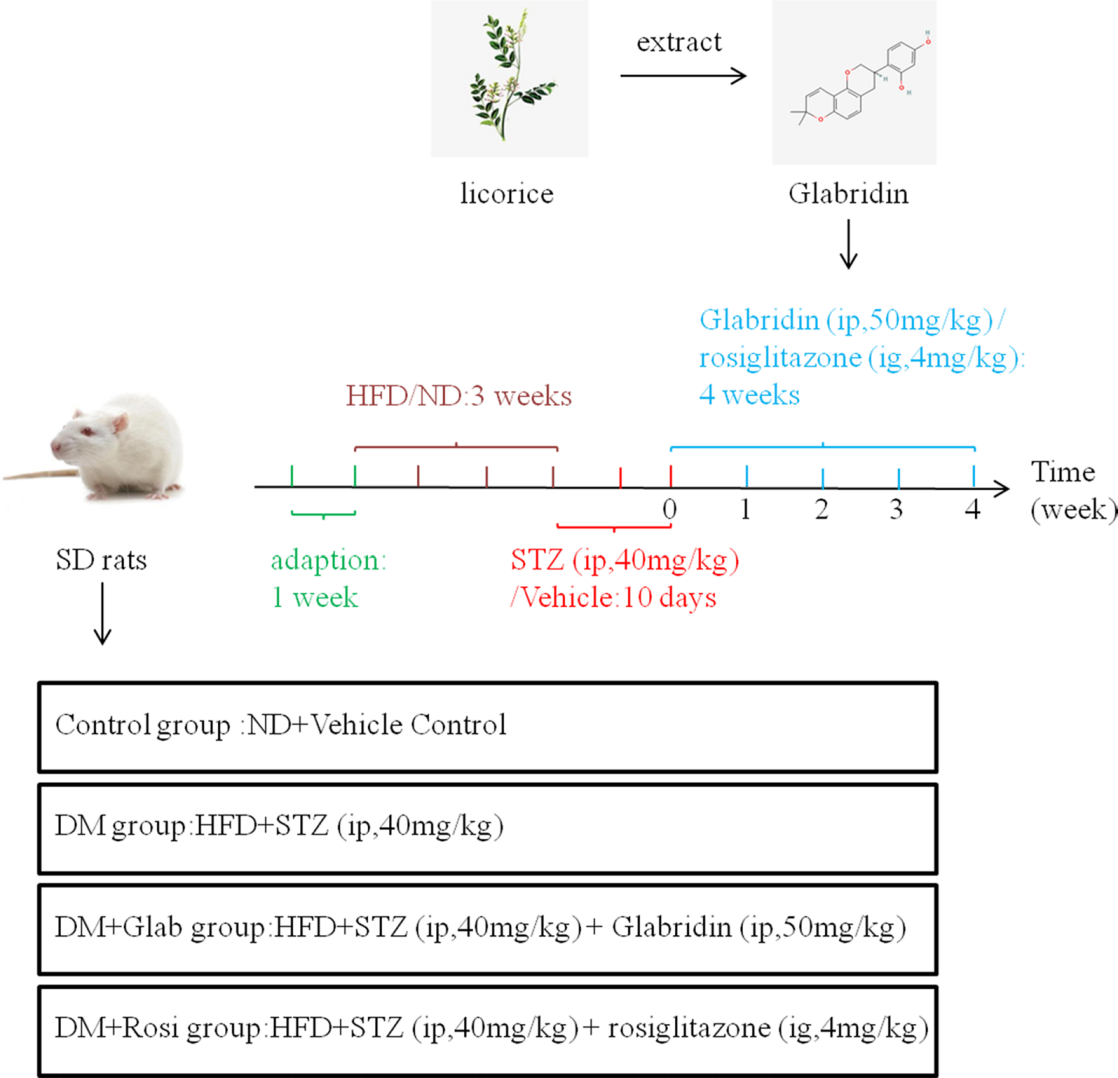
Animal experimental design. After adaptive feeding for a week, the DM model was established by a 3-week HFD followed 10-days intraperitoneal injection 40 mg/kg/day STZ. The control group (n = 5) received the citrated buffer only. DM model rats were randomly divided into three groups received diverse treatments: the DM group (n = 5), the DM + Glab group (n = 5), and the DM + Rosi group (n = 5). The latter two groups the corresponding treatments for a total of four weeks (from week 0 to week 4)

**Fig. 3:**
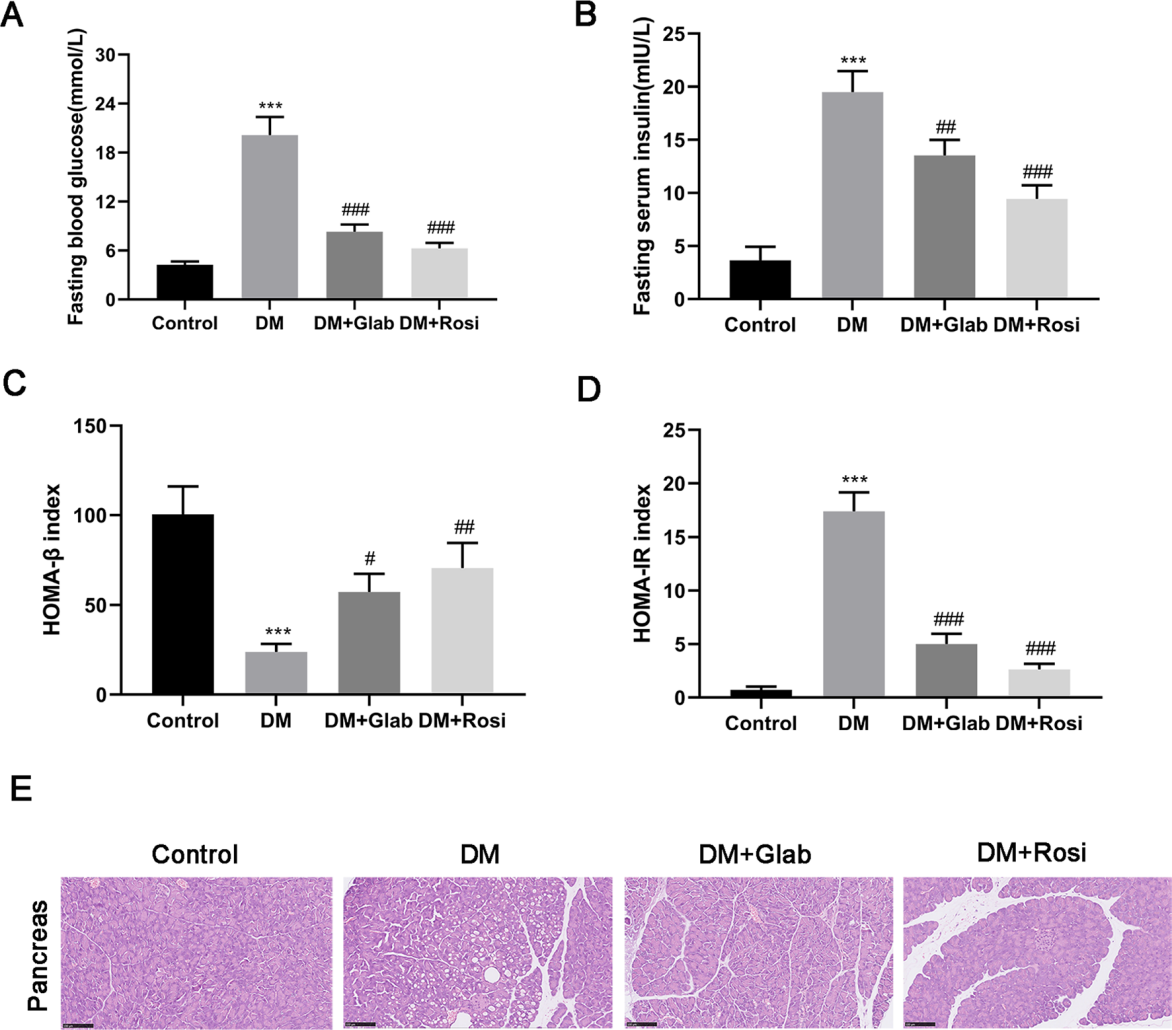
Glab attenuates diabetic symptoms of DM rats. **A** Fasting blood glucose (FBG) level. **B** Fasting insulin (FINS) level. **C** HOMA-β index. **D** HOMA-insulin index. **E** H&E staining of pancreatic tissues (magnification 200 × ; bar = 100 μm.). (^***^P < 0.005, vs. the control group; ^#^*P* < 0.05, ^##^*P* < 0.01, and ^###^*P* < 0.005, vs. the DM group)

The animal experimental design and groups were described in Fig. 2. The general states, which included body weight, food and water intake, were recorded once a week (Table 3). At the beginning of treatment, there was no obvious difference among the four experimental groups in the body weights, food and water intake of rats. From week 2 to week 4, the body weights of DM rats were continuously and significantly decreased compared to the control rats, while the food and water intake were significantly increased, indirectly confirming the successful establishment of the DM model. As expected, these manifestations could be effectively reversed by Glab treatment. Both the FBS and FINS of rats in the DM group were markedly elevated compared with those in the control group, which could be significantly reduced by Glab treatment (Fig. 3A, B). Moreover, Glab treatment also largely reversed the decreased HOMA-β index and increased HOMA-IR in the DM rats (Fig. 3C, D). In the H&E staining, numerous small vacuoles, wider interlobular and intralobular ducts were observed in the pancreas of DM rats (Fig. 3E). The abnormalities were restored by Glab treatments, which suggested Glab might also repair diabetes-induced damaged pancreatic architecture (Fig. 3E).

**Table 3:** Comparison of body weight, food intake and water intake of rats in different groups within 4 weeks

Group	n	0 W	1 W	2 W	3 W	4 W
Bodyweight(g)						
Control	6	241.38 ± 16.25	266.54 ± 21.49	304.75 ± 24.17	349.43 ± 25.71	385.64 ± 27.10
T2DM	6	243.95 ± 17.30	254.59 ± 20.85	259.36 ± 19.25^**^	246.58 ± 18.35^***^	218.15 ± 15.47^***^
T2DM + Glab	6	240.74 ± 15.85	257.13 ± 18.47	276.41 ± 20.63	297.75 ± 22.19^##^	324.58 ± 24.65^###^
T2DM + Rosi	6	245.26 ± 19.12	262.72 ± 21.05	287.19 ± 23.46^#^	316.33 ± 24.68^###^	347.83 ± 25.43^###^
Food intake(g/day)						
Control	6	19.31 ± 4.25	21.46 ± 4.72	22.31 ± 5.04	18.34 ± 4.58	20.12 ± 4.35
T2DM	6	18.75 ± 4.69	23.85 ± 5.15	29.48 ± 5.26^*^	36.64 ± 5.75^***^	42.19 ± 5.81^***^
T2DM + Glab	6	20.33 ± 5.12	22.48 ± 4.96	26.27 ± 5.09	27.13 ± 4.69^#^	24.71 ± 4.21^###^
T2DM + Rosi	6	19.42 ± 4.87	21.95 ± 5.10	24.43 ± 4.87	23.27 ± 4.25^##^	22.64 ± 3.95^###^
Water intake (mL/day)						
Control	6	45.73 ± 5.36	43.69 ± 4.73	46.29 ± 5.20	49.38 ± 5.95	45.28 ± 4.77
T2DM	6	47.24 ± 5.75	85.41 ± 7.29^***^	104.73 ± 9.84^***^	116.25 ± 12.84^***^	124.73 ± 15.46^***^
T2DM + Glab	6	44.39 ± 4.23	74.85 ± 6.24^#^	82.35 ± 8.55^##^	71.89 ± 6.73^###^	60.82 ± 6.25^###^
T2DM + Rosi	6	45.12 ± 4.87	64.37 ± 5.36^###^	75.24 ± 6.81^###^	62.38 ± 5.24^###^	55.60 ± 5.17^###^

*P < 0.05, **P < 0.01, and ***P < 0.001, vs. control; ^#^*P* < 0.05, ^##^*P* < 0.01, and ^###^*P* < 0.001, vs. T2DM

#### The protective effect of Glab on the kidneys in DN

**Fig. 4:**
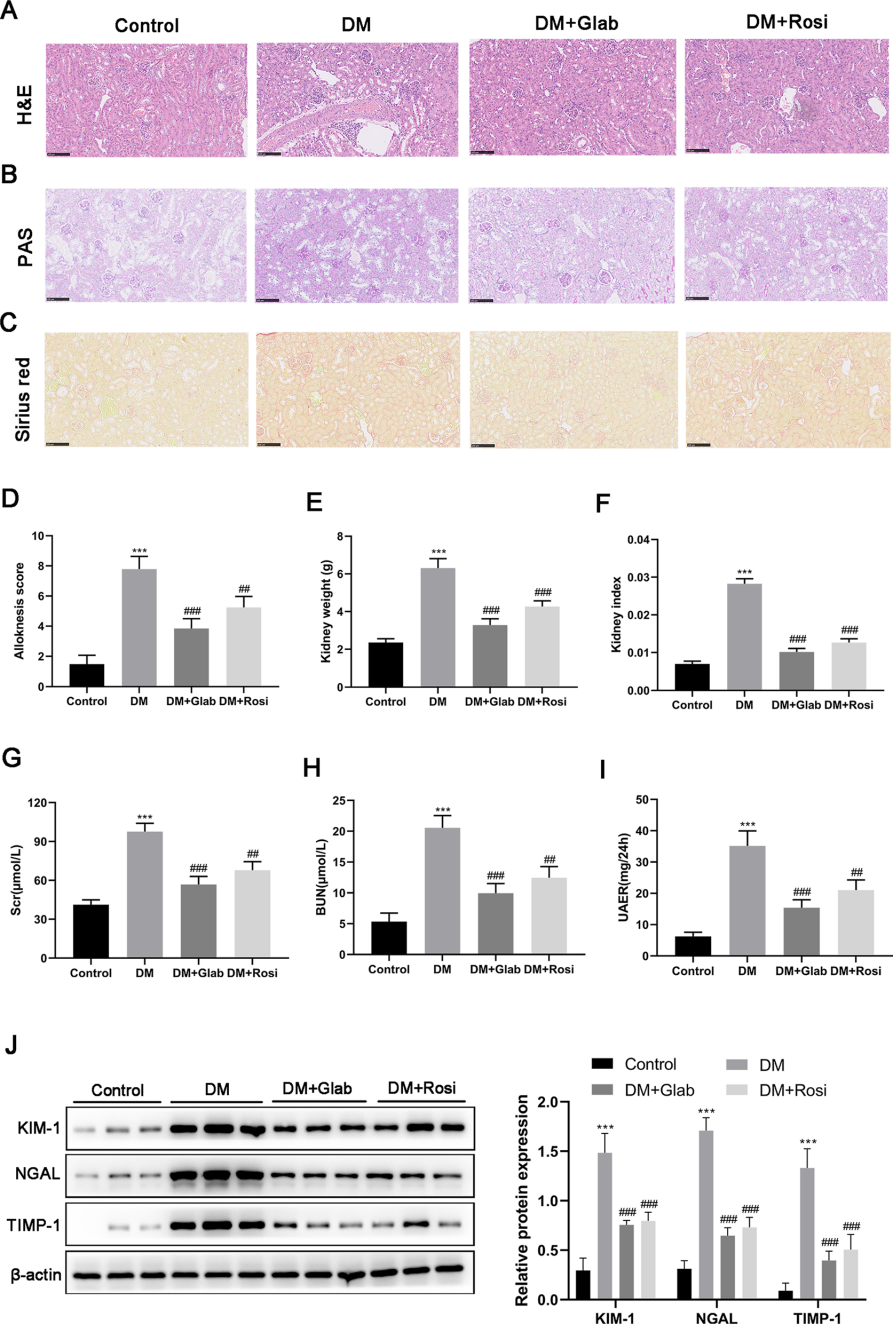
The effect of Glab on renal function. **A** H&E, **B** PAS, and **C** Sirius red staining of renal tissues (magnification 200 × ; bar = 100 μm). **D** Alloknesis score was recorded to evaluate mechanical itch behavior. **E** Kidney weight and (**F**) index. The renal function was assessed by detecting (**G**) serum creatinine (Scr), (**H**) blood urea nitrogen (BUN), and (**I**) urinary albumin excretion rate (UAER). **J** Kidney damage biomarkers (KIM-1, NGAL, and TIMP-1) in urinary excretion were detected by western blot (^***^P < 0.005, vs. the control group; ^##^*P* < 0.01 and ^###^*P* < 0.005, vs. the DM group)

It has been shown that the DM model induced by HFD combined with STZ injection will inevitably develop into DN. Thus, we further investigated the change in the structure and function of the kidneys from DM rats to explore whether Glab could ameliorate DN in vivo. In H&E and PAS staining, the kidneys of DM rats exhibited Bowman's capsule expansion, the mesangial and glomerular basement membrane thickening, and the mesangial cell hyperplasia (Fig. 4A, B). Besides, Sirius red staining revealed interstitial fibrosis in the kidneys of DM rats (Fig. 4C). All these histologic injuries could be partially relived following Glab treatment (Fig. 4A–C). The increased itch behavior and kidney index were also observed in DM rats, which were also reduced by Glab treatment (Fig. 4D–F). For renal function assessment, a significant increase of Scr, BUN, and UAER in DM rats demonstrated a remarkable renal dysfunction of rats induced by HFD/STZ (Fig. 4G–I), which confirmed rats with DN could be developed by HFD/STZ. After treating with Glab, the HFD/STZ -induced changes in these metabolites were markedly ameliorated (Fig. 4G–J). Moreover, the urinary kidney markers (KIM-1, NGAL, and TIMP-1) were also analyzed to verify the protective effect of Glab on renal function during the progression of DN. Compared with the control rats, DM rats showed a significant increase in the urinary excretion of KIM-1, NGAL, as well as TIMP-1, and Glab treatment reversed this alteration (Fig. 4J). These data collectively indicated that Glab indeed has protective roles against DN.

#### Effects of Glab on oxidative stress and ferroptosis in the kidneys of DM rats

**Fig. 5:**
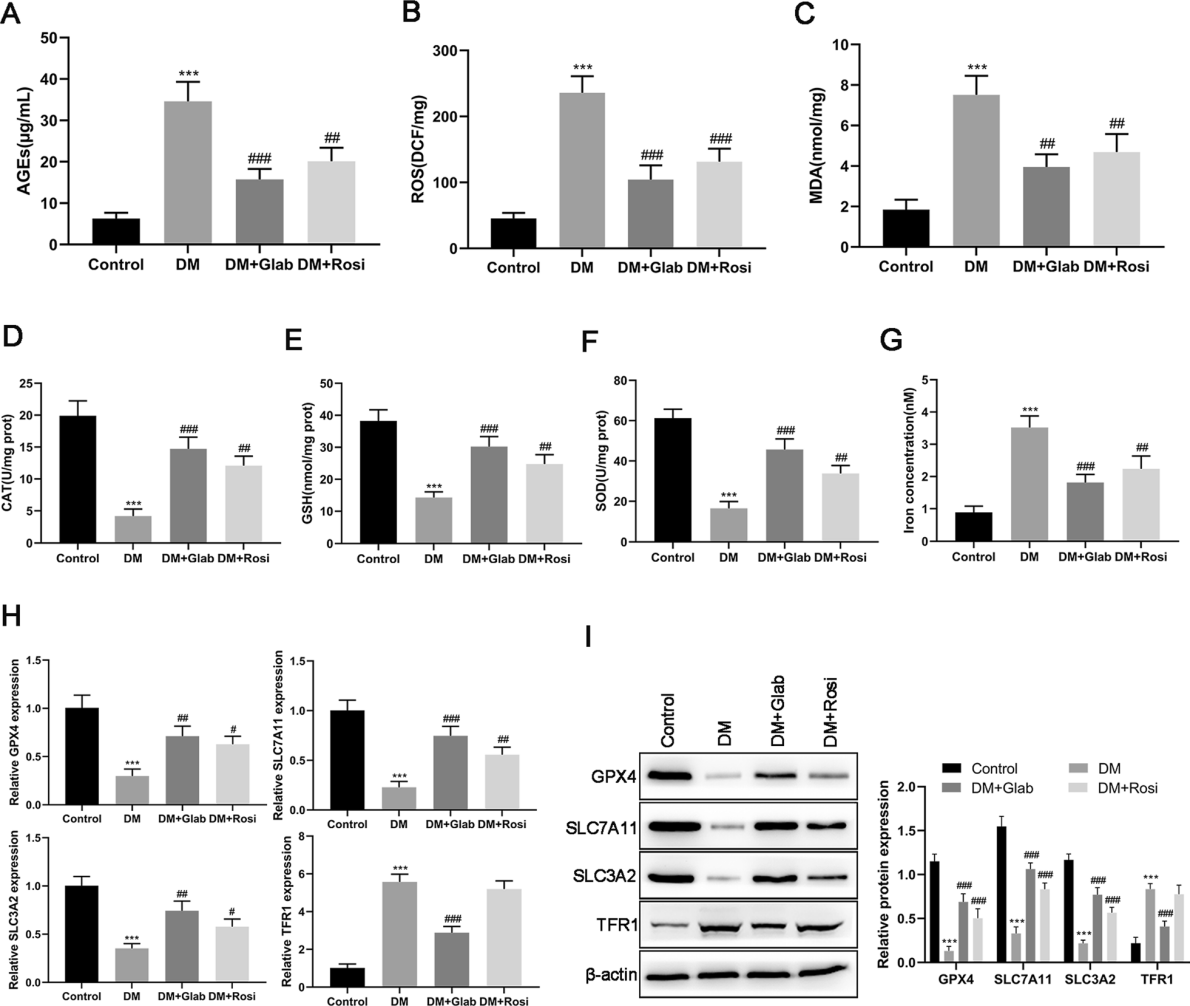
- Effects of Glab on the level of oxidative stress associated parameters, AGEs, and ferroptosis markers in the kidneys of the DM rats. The levels of (**A**) advanced glycation end products (AGEs), (**B**) reactive oxygen species (ROS), (**C**) malondialdehyde (MDA), (**D**) catalase (CAT), (**E**) glutathione (GSH), (**F**) superoxide dismutase (SOD), and (**G**) iron concentration determined by commercial kits. The expression of ferroptosis markers (GPX4, SLC7A11, SLC3A2, and TFR1) at (**H**) mRNA and (**I**) protein levels were detected by RT-qPCR and western blot, respectively (^***^P < 0.005, vs. the control group; ^##^*P* < 0.01 and ^###^*P* < 0.005, vs. the DM group)

The accumulation of AGEs in hyperglycemia has been implicated in the development of DN (Forbes et al. 2003). In our study, an obvious elevation of AGEs levels was observed in the kidneys of DM rats, which was reduced by the administration of Glab (Fig. 5A). In the meantime, ROS and MDA levels of the kidneys were also significantly increased in DM rats since the excessive production of AGEs could trigger oxidative stress (Fig. 5B, C). Moreover, the renal antioxidant systems, including CAT, GSH, and SOD, were obviously suppressed in DM rats (Fig. 5D, E). Under the intervention of Glab, the changes of the above parameters in DM rats were restored in different degrees (Fig. 5D–F). These results suggested that Glab might be a potent anti-oxidant that can relieve renal oxidative stress, thereby exerting a protective effect on the kidneys during DN development.

In addition to “response to oxidative stress”, our previous bioinformatics analysis revealed “response to metal ion” is also a biological process regulated by bioactive components of licorice. Previous studies demonstrated that ferroptosis was involved in DN and might be a promising therapeutic target for DN. Therefore, we further explored whether Glab exerts its role in DN by regulating ferroptosis. We found that iron content in the kidneys from DM rats was significantly higher than those from the control rats (Fig. 5G). Moreover, compared with the control group, a decrease in *GPX4*, *SLC7A11*, and *SLC3A2* expression with an increase in *TFR1* expression were also observed in DM rat kidneys (Fig. 5H, I), which further confirmed ferroptosis occurs in DM rat kidneys. Glab treatment showed an anti-ferroptosis effect on DM rat kidneys by reducing iron content and restoring the dysregulation of the above markers (Fig. 5G–I). The positive treatment group (Rosi) displayed a similar effect to Glab on oxidative stress and ferroptosis but did not influence both the mRNA and protein expression of TFR1 (Fig. 5).

#### Effects of Glab on oxidative stress and ferroptosis in HG-induced NRK-52E cells

**Fig. 6:**
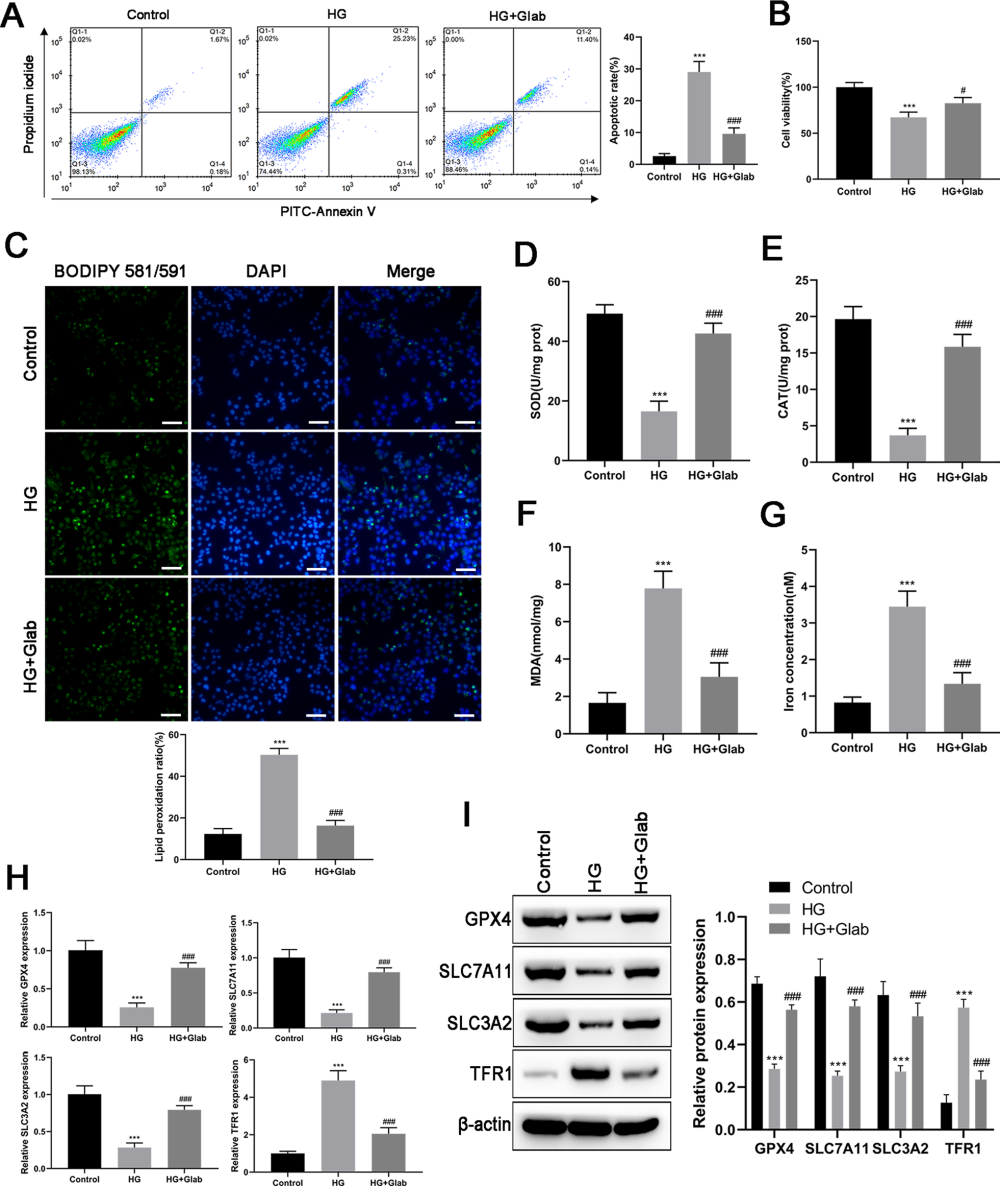
Effects of Glab on high glucose (HG) induced oxidative stress and ferroptosis in vitro. The condition of DN in NRK-52E cells was induced by 30 mM glucose. **A** Cell apoptosis was detected by annexin V-PI double staining. **B** Cell viability detected by CCK-8 assay. **C** Lipid peroxidation was determined by the fluorescent probe C11 BODIPY 581/591. Green and blue colors indicate peroxidated lipids and nucleus respectively. The levels of (**D**) SOD, (**E**) CAT, (**F**) MDA, as well as (**G**) iron concentration were measured by commercial kits. The expression of ferroptosis markers (GPX4, SLC7A11, SLC3A2, and TFR1) at (**H**) mRNA and (**I**) protein levels were detected by RT-qPCR and western blot, respectively (^***^P < 0.005, vs. the control group; ^#^*P* < 0.05 and ^###^*P* < 0.005, vs. the HG group)

To reinforce the observed findings in DM rats, NRK-52E cells were exposed to HG (30 mM glucose) to observe the effect of Glab on oxidative stress and ferroptosis in DN in vitro. The decreased cell viability and increased cell apoptosis were observed in NRK-52E cells after exposing HG (Fig. 6A, B), suggesting a injury of renal tubular epithelial cells induced by HG. Our data signed out that HG injures NRK-52E cells through triggering oxidative stress and ferroptosis, as demonstrated by the accumulation of lipid ROS products (Fig. 6C), MDA (Fig. 6F), and iron content (Fig. 6G), and the reduction of SOD (Fig. 6D) and CAT (Fig. 6E). The qRT-PCR and western blot analysis revealed the down-regulation of GPX4, SLC7A11, and SLC3A2, and up-regulation of TFR1 in NRK-52E cells upon HG situation (Fig. 6H, I). Glab treatment could effectively diminish the above alterations induced by HG in NRK-52E cells, which is in line with in vivo experimental data. In summary, in vivo and in vitro data indicated Glab had the potential of mitigating renal injury via suppressing ferroptosis.

### Glab regulates VEGF/Akt/ERK pathways in DN in vitro and in vivo

**Fig. 7:**
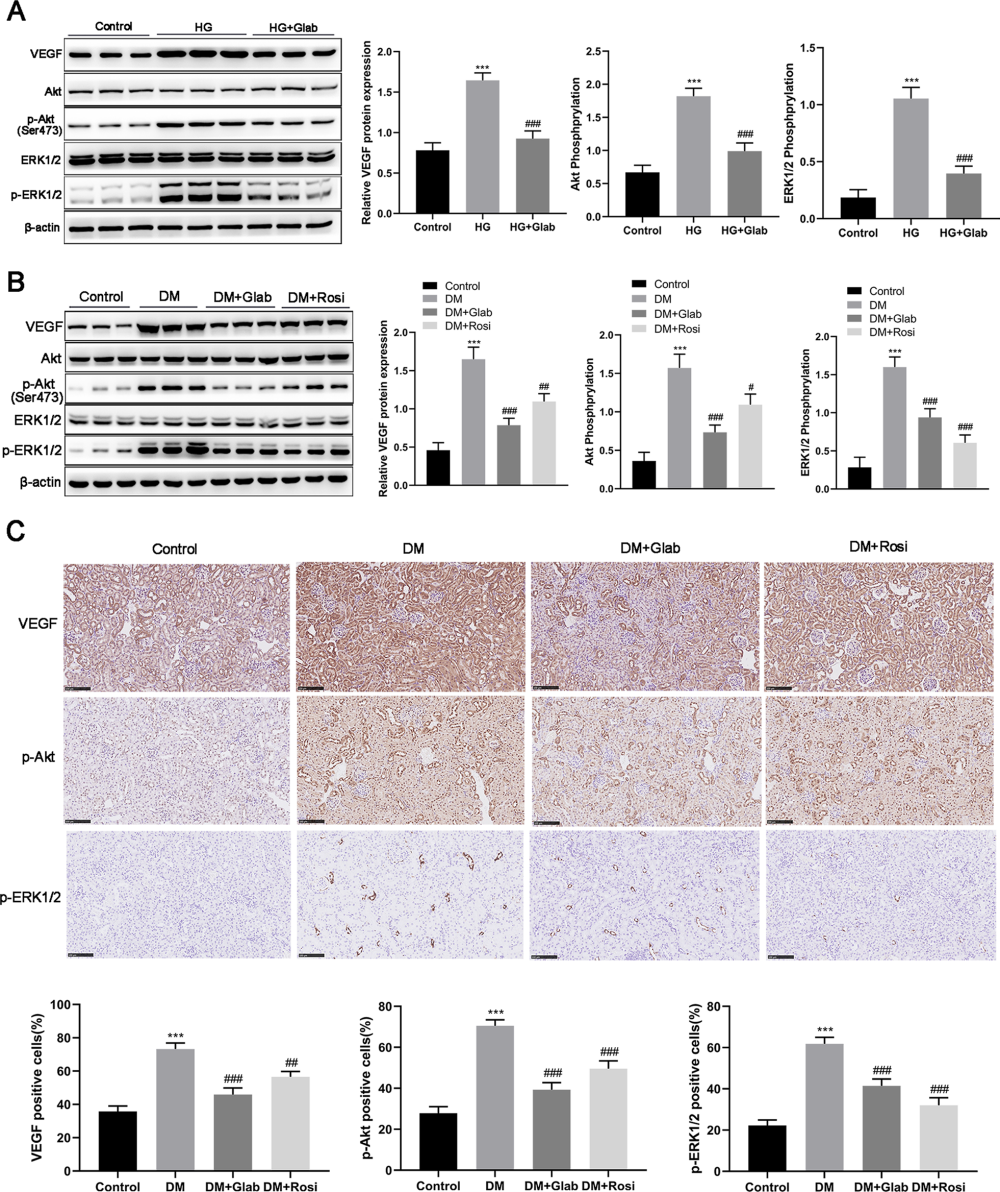
Effects of Glab on the VEGF/Akt/ERK pathway in DN in vitro and in vivo. Western blot was conducted to analyze the protein expression of VEGF, Akt, p-Akt, ERK1/2, and p-ERK1/2 in (**A**) HG-induced NRK-52E cells and (**B**) the kidney of DM rats. **C** Representative images and quantitation of immunohistochemistry for VEGF, p-Akt, and p-ERK1/2 in kidney tissues from rats of diverse groups (^***^P < 0.005, vs. the control group; ^#^*P* < 0.05, ^##^*P* < 0.01, and ^###^*P* < 0.005, vs. the DM group or the HG group)

In the consideration of our finding of previous bioinformatics analysis that 7 of 13 potential targets of Glab against DN were significantly associated with the VEGF signaling pathway (Fig. 1E), we further explore the regulatory role of Glab in the VEGF signaling pathway in DN. In vitro data showed a significant increase in not only VEGF expression but also Akt and ERK activation in HG cultured NRK-52E cells, which were partly repressed following Glab treatment (Fig. 7A). Expression changes of VEGF, Akt, p-Akt, ERK1/2, and p-ERK1/2 in vivo samples in the same tendency as in vitro experiment, supporting the suppressive effect of Glab on VEGF/Akt/ERK pathways in DN (Fig. 7B). Finally, we confirmed the expression of VEGF, p-Akt, and p-ERK1/2 by performing an IHC analysis. The results showed the number of cells positive for VEGF, p-Akt, and p-ERK1/2 was all obviously enhanced in the renal sections of DM rats when compared with the control rats (Fig. 7C). The increased number of cells positive for these proteins in the kidney of DM rats was largely diminished in the presence of Glab (Fig. 7C). Accordingly, Glab protects renal dysfunction from DN by suppressing ferroptosis, which might be dependent on the pathways of VEGF/Akt/ERK.

## Discussion

Glab is a bioactive component of licorice, which has been reported to exert hypoglycemic and protective effects on DM and its complications via anti-inflammation or antioxidative mechanism (Wu et al. 2013; Li et al. 2021). Herein, we revealed that Glab had the potential to treat DN by improving renal function through attenuating ferroptosis and regulating VEGF/Akt/ERK pathways.

It has been reported the protective action of licorice in DN in a previous study (Kataya et al. 2011), but little is known about the corresponding material bases and mechanisms. Our study proposed the network pharmacology analysis to screen out the putative bioactive components and targets against DN of licorice. The constructed network revealed that Glab is one of the main bioactive components of licorice against DN, which has potential research value in DN therapy. Thus, we explored the effect and underlying mechanism of Glab on renal damage by using HFD/STZ-induced DM rats and HG-induced NRK-52E cells in the subsequent experiments to explore the clinical potential of Glab.

HFD/STZ-induced model is widely used in the research on DN as HFD/STZ can cause IR and simultaneously destroy the islets of Langerhans to induce DM, thereby causing renal injury and even dysfunction (Kitada et al. 2016; Kundu et al. 2020). Consistent with a previous study (Wu et al. 2013), hyperglycemia of DM rats were effectively alleviated by Glab treatment as the increase of FBG and FINS levels was partly diminished. IR is a pathological condition that is characterized by a diminished ability of cells or tissues to respond normally to insulin, which could be quantified by HOMA-IR, and the β cell function could be assessed by HOMA-β (Wallace et al. 2004). Our data showed that Glab has the capacity of decreasing IR and restoring β cell function in DM rats. In addition, DM rats induced by HFD/STZ showed a significant bodyweight loss due to their prolonged hyperglycemia. Glab treatment attenuated the bodyweight loss of DM rats, indicating that Glab could protect the muscle tissue from hyperglycemia damage. Moreover, DM rats experienced a remarkable increase in kidney index in comparison to the control rats, which was also reversed following Glab treatment. As one of the common manifestations of DM patients, skin itching remains without specific medication (Horton et al. 2016). Increased itching behaviors were found in the HFD-rats after STZ injection, and the administration of Glab reduced this behavior, which suggested a potential role of Glab in relieving skin itching for DN patients.

During the progression of DM, renal dysfunctions occur due to injury in the kidney, which finally leads to DN. As known, Scr, BUN, and urinary albumin are the biochemical parameters for monitoring renal function (Dabla 2010). Due to the impaired glomerular filtration membrane, increased Scr, BUN, and urine albumin levels could be observed in DN patients. In many research, the alteration of these several biochemical parameters has been implicated in the progression of DN (Liang et al. 2017; Ma et al. 2013). Hence, SCr, BUN, and UAER levels of the rats were monitored in this study. In line with previous experimental evidence, DM rats induced by HFD/STZ manifested typical renal dysfunction of DN, as demonstrated by a significant increase level of SCr, BUN, and UAER. Glab treatment effectively diminished these changes in renal function, suggesting the renoprotective effect of Glab against renal dysfunction in DN. Moreover, this finding was confirmed by the pathological analysis, as confirmed by amelioration in renal tissue pathological changes and renal fibrosis. Recently, multiple urinary kidney biomarkers were reported to assess tubular and glomerular damage (Lee et al. 2017; Cheon et al. 2016). KIM-1 is a known marker of kidney tubular injury, of which expression is elevated during DN in DM patients (Khan et al. 2019; El-Ashmawy et al. 2015). NGAL is also another marker of renal injury with high prognostic and diagnostic value in DN (Kim et al. 2012), which exhibits an iron-dependent biological activity (Kuwabara et al. 2009). TIMP-1 has been reported to elevate in chronic renal disease patients, which could promote tubulointerstitial fibrosis and regulate extracellular matrix synthesis and degradation (Sieber et al. 2009; Chromek et al. 2004). In this study, urinary excretion levels of KIM-1, NGAL, and TIMP-1 were dramatically elevated in DM rats, and treating with Glab markedly alleviated this elevation. These results further corroborated our finding that Glab is capable of protecting the kidney from dysfunction induced by sustained hyperglycemia.

Despite the precise pathological mechanism under DN being largely unclear, accumulating evidence supported that oxidative stress is a decisive factor of DN progression (Kashihara et al. 2010). The accumulation of AGEs and increased ROS production resulting from sustained hyperglycemia could lead to renal cell injury, thereby causing renal dysfunction (Yamagishi and Matsui 2010). Previous animal experiments demonstrated multiple natural substances can attenuate diabetic renal pathological changes and renal dysfunction by exerting an anti-oxidize effect (Kundu et al. 2020; Sohn et al. 2013). Similar to the previous studies, high concentrations of AGEs were observed in the kidney of DM rats. This condition was sharply reversed by Glab. Moreover, increasing ROS and MDA levels and decreasing CAT and SOD activities in both DM rat kidneys and HG-induced NRK-52E cells were also largely reversed after the treatment of Glab. All these data indicated a potent effect of Glab in suppressing oxidative stress in DN. Ferroptosis is a type of cell death resulting from ROS-mediated lipid peroxidation, which has been implicated in the progression of DN in recent years (Wang et al. 2020; Li et al. 2021). In consideration of the vital role of iron in the process of ferroptosis, we monitored the change of iron concentration in rat kidneys and NRK-52E cells after HFD/STZ or HG stimulation, and found ferroptosis occurrence during DN. This finding was further verified by the up-regulation of *TFR1* and down-regulation of *GPX4*, *SLC7A11*, and *SLC3A2* in DM rat kidneys and HG-induced NRK-52E cells. As known, both *SLC7A11* and *SLC3A2* are members of the solute carrier family, which are two subunits of an X_C_^¯^ system that controls the cellular entry and exit of cystine and glutamate, respectively (Lewerenz et al. 2013). The downregulation of *SLC7A11* and *SLC3A2* affects GSH synthesis by decreasing the absorption of cystine mediated by system X_C_^¯^, leading to a decline in GPX4 activity, an impairment in cell antioxidant capacities, lipid ROS accumulation, and ultimately the occurrence of oxidative damage in renal endothelial cells, thereby accelerating the progression of DN (Zhang and Li 2022; Singh et al. 2011). Surprisingly, our study showed that Glab treatment could effectively repress ferroptosis by reversing the downregulation of *SLC7A11* and *SLC3A2* both in vitro and in vivo. Thus, these data collectively revealed that Glab might be capable of antagonizing oxidative stress and ferroptosis in DN.

Finally, we preliminarily explore the mechanism behind the role of Glab in DN. Based on the result of bioinformatics analysis, we speculated that the VEGF pathway might be attributed to the effect of Glab in DN. Notably, our in vitro and in vivo experiments validated this speculation. It is widely accepted that the abnormal expression of VEGF is closely related to the development of diabetic microvascular complications (Tremolada et al. 2007). Mounting evidence is available on the causative role of VEGF in the pathogenesis of NP (Tufro and Veron 2012; Cha et al. 2004). Vriese et al. (Vriese et al. 2001) demonstrated that the treatment with the antibody of VEGF could ameliorate hyperfiltration, albuminuria, and glomerular hypertrophy in HFD/STZ-induced DM rats, suggesting VEGF could be a target for therapeutic intervention in DN. Additionally, the Akt and ERK1/2 signaling pathways were proven to be associated with DN (Xu et al. 2019). It has been reported that suppressing Akt could repress HG-induced inflammation and ECM procedure in mesangial cells (Xu et al. 2019). Multiple studies observed increased p-ERK1/2-mediated hypertrophy and extracellular matrix accumulation in the DM model in vivo and in vitro (Mage et al. 2002; Fujita et al. 2004). Our data showed that the expression of VEGF was significantly increased in diabetic kidneys and HG-induced NRK-52E cells, which is consistent with previous reports, whereas Glab treatment reversed these alterations, Thus, the suppression of the VEGF/Akt/ERK pathways by Glab could, at least in part, contributes to its protective effect on the diabetic kidney.

The current study revealed that Glab treatment could alleviative hyperglycemia, IR, and renal dysfunction in vivo, and repress oxidative stress, ferroptosis, as well as the VEGF/Akt/ERK pathways both in vivo and in vitro. However, this study has some limitations that should be addressed. First, the role of VEGF/Akt/ERK pathways in the protective effects of Glab is not elucidated sufficiently. Therefore, the effects of Glab on DN are required to be verified after blocking these pathways by the specific inhibitors in the future. Further, the specific binding between Glab and molecules of these pathways is a focus of our future study, which could be identified based on a technique of drug affinity responsive target stability. Besides, whether the protective effect of Glab on DN is attributed to its anti-diabetic or antioxidant effects or the combination of them is still unclear; hence, exploring the effect of Glab on a non-diabetic model of nephropathy is another focus of our future study.

## Conclusions

In summary, our study, for the first time, demonstrated a certain degree of therapeutic effects of Glab on the damage, dysfunction, and oxidative stress of the kidney in DN. The potential underlying mechanism is likely to mitigate ferroptosis and regulate the VEGF/Akt/ERK pathways, which remains required to be verified in future studies. Our findings provide scientific information on Glab serves as a therapeutic candidate for effectively alleviating renal function damage in DN.

## Data Availability

Not applicable.

## Abbreviations

Glab: Glabridin
DN: Diabetic nephropathy
DM: Diabetes mellitus
ESRD: End-stage renal disease
TCM: Traditional Chinese medicine
GO: Gene Ontology
BP: Biological process
CC: Cell component
MF: Molecular function
KEGG: Kyoto Encyclopedia of Genes and Genomes
SD: Sprague–Dawley
HFD: High-fat diet
ND: Normal diet
FBG: Fasting blood glucose
FINS: Fasting insulin
ELISA: Enzyme linked immunosorbent assay
HOMA-β: Homeostasis model assessments of β-cell function
HOMA-IR: Homeostasis model assessments of insulin resistance
Scr: Serum creatinine
BUN: Blood urea nitrogen
UAER: Urinary albumin excretion rate
H&E: Hematoxylin–eosin
PAS: Periodic acid-Schiff
AGEs: Advanced glycation end products
SOD: Superoxide dismutase
CAT: Catalase
GSH: Contents of glutathione
MDA: Malondialdehyde
ROS: Reactive oxygen species
qRT-PCR: Quantitative real-time PCR
